# The Tendency of Medical Education

**Published:** 1913-09-06

**Authors:** 


### The Hospital

The Workers' Newspaper of Administrative Medicine and Institutional Life, Administration, National Insurance and Health.

No. 1419, Vol. LIV. SATURDAY, SEPTEMBER 6, 1913. PRICE ONE PENNY

### EDUCATIONAL NUMBER.

### THE TENDENCY OF MEDICAL EDUCATION.

The growth and development of medical science during the last thirty or forty years—in fact, since the rise of Listerian surgery and of bacteriology—has been both luxuriant and full of previously unsuspected variations. From time to time the importance of many new special branches of science has been recognised by their inclusion in the curriculum of study which is demanded of those who would qualify as registered medical practitioners. To train students in those new sciences, laboratories and lecture-rooms and teachers have had to be provided; and in a great number of respects medical education is quite different to what it was a quarter of a century ago. In principle, however, the difference is not so great as it would seem to be from a cursory survey of the details of what the modern medical student has to apply himself to. When once the old apprenticeship system of medical training was abandoned, the hospital-medical-school system which supplanted it maintained a tradition of continuous evolution without any sudden or violent transition stages. To this day the various centres of medical education (such of them, that is, as have a past history of any considerable length) are distinguishable each by certain peculiarities which inevitably affect the alumni proceeding from them into the various branches of practice.

Just at the present time, as most medical men are aware, a hot controversy is going on over the future of medical education as far as London University is concerned. We have dealt with some of the principal points at issue in this debate recently (The Hospital, August 23, 1913, p. 609), and there is no need for us to repeat here the views then expressed. It is, however, not altogether superfluous to take a rather broader view of British medical education and its tendencies; for the conditions which prevail are in many ways exceptional, and different to those in other centres of medical training. In London, clinical material—that is, patients of the hospital class—is far and away more abundant than in any other city in the world. Indeed, it is so abundant that it constitutes almost an embarrassment of riches to those who have to make use of it for purposes of medical education. In consequence, the London student's opportunities of practical instruction by personal observation and by strictly clinical teaching, as well as by the responsibilities of resident medical office, are far greater and more valuable than those anywhere else in Great Britain. On the other hand, the chances and emoluments of consulting practice are so enormously greater in London than elsewhere that they inevitably attract the best intellects among the hospital-medical-school teachers, who attain much larger incomes by this work than they can possibly hope to do by teaching. Thus the younger teachers, who have not yet attained much fame and success as consultants, do most of the real work of medical education in London.

The result of these two peculiarities of the Metropolis is that the actual standard and volume of formal lecturing by experienced teachers is lower than in some other places, for example, in Edinburgh; and medical students are much less spoon-fed in consequence. But another result is that the London students of average ability and reasonable diligence are turned out better equipped practically than those of most of the other schools. This fact is unquestionable in our opinion, a conviction based upon contact with many men from many schools. The explanation given above may or may not be the correct one, but it seems a reasonable inference from the observed facts. It is, on the other hand, to be admitted that medical education, both in London and the provinces, might be better than it is. The particular remedies propounded by the Haldane Commission at Mr. Flexner's instance are, for the reasons given a fortnight ago, in our judgment, unreasonable; but at the same time it is easy enough to agree that the teaching of students ought to be a better paid and less casual occupation than it certainly is in London to-day. To remedy this weakness without disturbing the peculiar efficiency of London medical education based upon the quantity and quality of the clinical material available may be difficult, but should not be impossible.

In other respects the past year has seen few innovations and changes in medical education. One instance is afforded by the alteration of the syllabus for the Primary examination for the F.R.C.S. (England). Instead of one paper in physiology and one in anatomy, there will be two in each subject; but candidates, instead of answering all the questions in each paper, will only be allowed to select half of them. This should serve to diminish the distinct element of flukiness which has long marred the reputation of this exceedingly and unnecessarily severe examination. In respect of post-graduate education, which is gradually receiving a measure of the respect and attention due to it, the outstanding features of the year are two in number: the death of Sir Jonathan Hutchinson, founder and ever-generous patron of the Polyclinic, and the new buildings of the London School of Tropical Medicine, which will enhance the magnificent reputation which that school has obtained throughout the world.

